# Flexible Polymer-Stabilized Liquid Crystal Films Based on Radical-Promoted Cationic Co-Polymerization of Epoxy Monomers for Smart Windows

**DOI:** 10.3390/polym18131675

**Published:** 2026-07-07

**Authors:** Bingxuan Wang, Tianfu Zhou, Jiayu Li, Yingjie Shi, Meiqi Yang, Yuxin Qian, Yanzi Gao, Meina Yu, Cheng Zou, Yuanwei Chen, Huai Yang

**Affiliations:** 1 Institute for Advanced Materials and Technology, University of Science and Technology Beijing, Beijing 100083, China; d202310751@xs.ustb.edu.cn (B.W.); 18579178779@163.com (T.Z.); 13131466876@163.com (J.L.); syj0625@163.com (Y.S.); yangmeiqi32@163.com (M.Y.); qianyuxin0911@163.com (Y.Q.); gaoyanzi@ustb.edu.cn (Y.G.); yumeina@ustb.edu.cn (M.Y.); 2 School of Materials Science and Engineering, Peking University, Beijing 100083, China; yanghuai@pku.edu.cn

**Keywords:** polymer-stabilized liquid crystals, epoxy resin, smart windows, electro-optical properties, peel strength

## Abstract

Polymer-stabilized liquid crystal (PSLC) films are promising for smart window applications because of their transparent-to-scattering switching behavior. However, conventional acrylate-based PSLC films often suffer from poor mechanical robustness and weak interfacial adhesion, limiting their use in flexible devices. Herein, epoxy-based PSLC films have been prepared through radical-promoted cationic photopolymerization using a difunctional epoxy monomer, E6M, and a series of liquid-crystalline monoepoxy monomers, E-nOCB. The effects of alkyl chain parity, chain length, and E6M/E-10OCB ratio on polymer morphology, electro-optical behavior, and peel strength were systematically investigated. Even-numbered E-nOCB monomers favored the formation of regular columnar polymer structures and improved optical contrast, whereas odd-numbered monomers produced more disordered networks with higher peel strength. Among them, the sample prepared with E-10OCB showed a better balance between electro-optical performance and mechanical adhesion. At a fixed total polymer content of 15 wt%, optimizing the E6M/E-10OCB ratio enabled the sample doped with E-10OCB to achieve the highest contrast ratio of 160.91 while increasing the peel strength from 47.28 to 55.69 kPa compared with the sample without E-10nOCB. These results demonstrate that regulating monoepoxy/diepoxy composition and alkyl chain structure is an effective strategy for improving the overall performance of epoxy-based PSLC films for smart windows.

## 1. Introduction

Increasing energy demand and the need to reduce building-related carbon emissions have stimulated the development of smart window technologies [1,2,3]. Among various electrically switchable light-control systems, liquid crystal/polymer composite films are attractive because of their fast response, low power consumption, and durability, which are beneficial for large-area optical modulation [4,5]. According to their internal structures and switching modes, electrically controlled LC films are commonly classified into polymer-dispersed liquid crystal (PDLC) films and polymer-stabilized liquid crystal (PSLC) films [6,7]. PDLC films are usually opaque in the off state and become transparent under an applied voltage, whereas PSLC films show the opposite switching behavior. In a typical PSLC film, vertically aligned LC molecules give a transparent off state. When an electric field is applied, the LC molecules reorient, producing a refractive-index mismatch between the LC and polymer phases and thereby generating strong light scattering. This transparent-to-scattering switching mode makes PSLC films promising for privacy protection and energy-saving applications [8].

In recent years, thiol–acrylate and thiol–vinyl ether systems have been introduced to regulate polymerization kinetics, phase separation, and polymer-network morphology in LC/polymer composite films [9]. For example, thiol–acrylate chemistry has been used to construct reverse-mode PSLC films [10], while thiol–vinyl ether systems have been developed for electrically controlled dimming films and PDLC composite films with low driving voltage and high contrast ratio [11]. Despite these advantages, the practical use of PSLC films is still limited by the properties of their polymer networks. Conventional PSLC films are mainly prepared from acrylate-based monomers through UV-initiated free-radical polymerization [12,13]. The resulting crosslinked polymer networks can stabilize LC alignment and provide fast optical switching, but their relatively high elastic modulus and limited fracture toughness often lead to brittleness, stress-induced cracking, and weak interfacial adhesion under bending or tensile deformation [14]. These limitations are particularly unfavorable for flexible smart windows, where the polymer matrix must maintain both LC alignment and mechanical integrity during repeated deformation [15].

Epoxy resins provide a possible route to address this problem because of their strong adhesion, corrosion resistance, and mechanical robustness, and they have been widely used in encapsulation, coatings, and protective materials [16,17,18,19,20]. In liquid crystal/polymer composite films, epoxy-based networks can also confine liquid crystal domains and regulate electro-optical behavior through their network density and phase-separated morphology. Shen et al. studied the relationship between fabrication conditions and electro-optical properties in epoxy resin-based PSLC films [21]. They showed that the effects of the liquid crystalline epoxide content, UV intensity, polymerization temperature, and initiator content strongly affected polymer morphology and electro-optical behavior. Chen et al. also reported an epoxy polymer-stabilized LC composite for energy-efficient smart windows [22]. The device retained the reverse-mode switching behavior of PSLC films and showed improved stability. Wu et al. prepared acrylate/epoxy resin-based PSLC films by stepwise photopolymerization [23]. Their results showed that polymer content and acrylate/epoxy composition played important roles in determining polymer morphology and electro-optical performance. More recently, Song et al. improved both the electro-optical properties and peel strength of epoxy-based PSLC films by regulating epoxy-monomer composition and polymer-network morphology [24].

However, epoxy-based PSLC systems still face several challenges. Cationic photopolymerization of epoxy groups is usually slower than free-radical polymerization, which may limit rapid film fabrication. In addition, the electro-optical performance and peel strength of epoxy-based PSLC films are strongly affected by the polymer morphology, which depends sensitively on monomer functionality, chain structure, and formulation. Therefore, simply replacing acrylate monomers with epoxy monomers is not sufficient; a rational design of the epoxy network is required to balance optical contrast, driving voltage, peel strength, and flexibility.

In this work, a radical-promoted cationic photopolymerization strategy was used to construct epoxy-based PSLC films. A difunctional epoxy monomer, E6M, was combined with a series of liquid-crystalline monoepoxy monomers, E-nOCB, to regulate the polymer network structure. The influence of alkyl chain parity and chain length was first examined using E-nOCB monomers with different spacer lengths. After identifying E-10OCB as a suitable monoepoxy component, the E6M/E-10OCB ratio was further varied at different total polymer contents to clarify how monomer composition controls polymer morphology, liquid crystal alignment, electro-optical switching, and peel strength. Sample C2 achieved the highest contrast ratio of 160.91, indicating that an appropriate network-forming tendency and polymer morphology are essential for efficient transparent-to-scattering switching. This study provides a formulation-based strategy for improving the balance between optical modulation and mechanical reliability in epoxy-based PSLC films.

## 2. Materials and Methods

### 2.1. Materials

**Figure 1:**
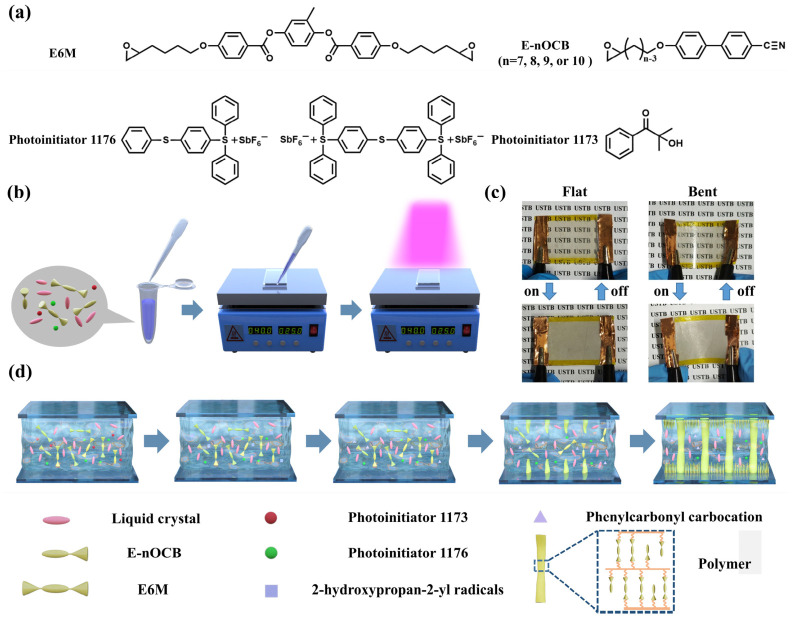
(**a**) Molecular structures of E6M, E-nOCB, photoinitiator 1173, and photoinitiator 1176 used in this study. (**b**) Schematic illustration of the sample preparation process. (**c**) Photographs of the PSLC film under bending in the off and on states. (**d**) Schematic illustration of the formation of the polymer network during UV-induced polymerization.

The nematic liquid crystal with negative dielectric anisotropy used in this study was GXV-7822-180 (T_NI_ = 380.15 K), which was purchased from Yantai Xianhua Technology Group Co., Ltd. (Yantai, China). The difunctional epoxy monomer used in the experiments was E6M (4′-epoxycyclohexylmethyl 3,4-epoxycyclohexane carboxylate), which was purchased from Nanjing Shuxin Science and Technology Co., Ltd. (Nanjing, China). Photoinitiators 1173 and 1176 were purchased from Shanghai Aladdin Biochemical Technology Co., Ltd. (Shanghai, China). E-nOCB monomers (*n* = 7–10) and their synthetic intermediates V-nOCB (*n* = 7–10) were synthesized according to the procedures described in Figure S1 in the Supporting Information. All reactants and reagents used for the synthesis were purchased from Anhui Senrise Technologies Co., Ltd. (Shanghai, China). The molecular structures of the monomers and photoinitiators used in this study are illustrated in Figure 1a.

### 2.2. Sample Preparation and Working Mechanism

The sample preparation procedure is illustrated in Figure 1b. The liquid crystal, photoinitiator 1173, photoinitiator 1176, and epoxy monomers were first mixed in a centrifuge tube at predetermined ratios. The mixture was then heated with hot air from a hairdryer and homogenized using an oscillator. Subsequently, it was introduced into a liquid crystal cell whose inner surfaces were coated with indium tin oxide (ITO) electrodes and a polyimide alignment layer for vertical alignment. Driven by capillary action, the mixture filled the gap between the two conductive substrates, which was fixed at 20 μm by spacers. After the cell was filled, the sample was exposed to UV light (365 nm, irradiation area: 100 × 100 mm; Yuntong Technology, Shanghai, China) to induce photopolymerization. The photopolymerization procedure is illustrated in Figure 1d. During this process, polymerization of the epoxy monomers triggered phase separation between the liquid crystal and polymer phases, resulting in the formation of a polymer network. Figure 1c shows photographs of the PSLC film in the off and on states. In the off state, the vertically aligned liquid crystal molecules rendered the film transparent in both flat and bent configurations. Liquid crystals with negative dielectric anisotropy were used to enable the reverse-mode switching behavior of the PSLC films. Since the electric field is applied approximately perpendicular to the substrates, the director of a negative-dielectric-anisotropy liquid crystal tends to orient perpendicular to the field direction under an applied voltage. This drives the initially vertically aligned liquid crystal molecules to tilt away from the substrate normal, thereby increasing the refractive-index mismatch between the liquid crystal and polymer phases and producing strong light scattering in the on state. The film also showed visible switching behavior under a bent configuration.

### 2.3. Characterization

The chemical structures of the synthesized V-nOCB and E-nOCB monomers were characterized by nuclear magnetic resonance spectroscopy (NMR, AVANCE III 400 MHz, Bruker, Ettlingen, Germany) and Fourier-transform infrared spectroscopy (FTIR, Spectrum 100, PerkinElmer, Shelton, CT, USA). The phase-transition temperatures of the LC monomers and reverse-mode dimming films were determined by differential scanning calorimetry (DSC; DSC 8000, PerkinElmer, USA) at a heating rate of 10 °C min^−1^.

The electro-optical properties of the samples were characterized using a Liquid Crystal Comprehensive Parameter Tester (LCT-P1000, Changchun North Liquid Crystal Engineering Research and Development Center Co., Ltd., Changchun, China). A halogen light source with a wavelength centered at 560 nm was used as the incident light source, and a square-wave driving voltage with frame inversion at 100 Hz was applied during the measurements. The transmitted light passing through the sample was detected by a photodiode detector, with the collection angle controlled within ±2°. Prior to testing, air was used as the reference to normalize the transmittance of the samples. The transmittance under the applied voltage was measured in the ballistic light mode.

The morphology of the polymer network was characterized by scanning electron microscopy (SEM, HITACHI S-4800, Hitachi, Tokyo, Japan). Prior to SEM observation, the samples were cut into small pieces and immersed in cyclohexane for 4 days, with the solvent replaced daily to ensure the complete removal of the liquid crystal molecules [25,26]. The samples were then dried in a vacuum oven at 60 °C for 12 h and sputter-coated with gold before SEM observation. A polarized optical microscope (POM, Nikon LV100N POL, Nikon, Tokyo, Japan) was used to observe the textures and alignment of the reverse-mode dimming films.

The peel strength of the PSLC films was measured using an electronic universal testing machine (CMT4503, MTS Industrial Systems Co., Ltd., Shenzhen Branch, Shenzhen, China). The two protruding ends of each sample were vertically clamped between the two grips of the testing machine, with the sample’s center line aligned with the loading axis to ensure uniform force loading. Sandpaper was placed between the grips and the sample to increase friction and prevent slipping during the test. The stretching rate was set to 5 mm min^−1^, and the effective bonded area of the sample was 2 mm × 3 mm. The peel strength was calculated from the measured force and the effective bonded area.

## 3. Results and Discussion

To investigate the effects of epoxy monomers with different alkyl chain lengths on the electro-optical performance of PSLCs and the polymer morphology formed within the liquid crystal cell, a series of liquid-crystalline epoxy monomers (E-nOCB, *n* = 7, 8, 9, or 10) were designed, synthesized, and confirmed by NMR and FTIR spectra, as shown in Figures S2–S7. The corresponding samples were prepared with different monoepoxy monomers for characterization. In these samples, the total content of the diepoxy monomer E6M and the monoepoxy monomer E-nOCB was fixed at 20 wt%. The sample compositions and polymerization conditions are listed in Table 1.

**Figure 2:**
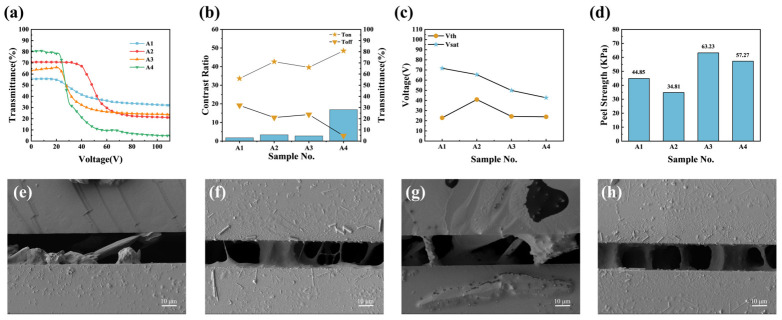
Electro-optical and mechanical performance when PSLC films prepared with epoxy monomers E-nOCB of different alkyl chain lengths: (**a**) transmittance-voltage curves, (**b**) contrast ratios and transmittance, (**c**) driving voltage, and (**d**) peel strength of Samples A1-A4; (**e**–**h**) SEM images of (**e**) A1, (**f**) A2, (**g**) A3, and (**h**) A4. All the scale bars are 10 μm.

As shown in Figure 2a,b, when the total content of E6M and E-nOCB was fixed at 20 wt%, the samples exhibited a pronounced odd-even effect in their electro-optical behavior. Compared with the samples containing E-7OCB and E-9OCB, those containing the even-numbered chain monomers E-8OCB and E-10OCB showed higher contrast ratios. This difference may be related to differences in the packing characteristics of the odd- and even-numbered alkyl chains. As shown in Figure 2e–h, the odd-numbered chains tended to form a more disordered polymer morphology with poorer vertical alignment, whereas the even-numbered chains more readily formed well-defined columnar polymer structures. For the E-nOCB series, both the odd- and even-numbered homologues exhibit nematic liquid-crystalline behavior and are incorporated into the polymer network during photopolymerization. Therefore, the odd-even difference observed in the SEM images should be mainly attributed to the effect of alkyl-chain parity on molecular conformation and packing during network formation. The odd-numbered alkyl chains tend to induce a more bent molecular conformation, which may disturb regular packing and result in a more disordered polymer morphology. In contrast, the even-numbered alkyl chains favor a relatively more extended conformation, facilitating more ordered packing and the formation of columnar-like polymer structures.

For the odd-numbered-chain samples, the disordered polymer network disrupted the uniform vertical alignment of the liquid crystal molecules in the off state, leading to local lateral or tilted orientations and thus a lower off-state transmittance, as shown in Figure 2b. In addition, the relatively loose and disordered polymer network exerted weaker anchoring on the liquid crystal molecules, allowing them to reorient under a lower applied voltage and thus resulting in a lower driving voltage. However, the nonuniform network structure also reduced the consistency of field-induced molecular reorientation, leading to inferior electro-optical switching behavior.

In contrast, the more regular columnar polymer structures in the even-numbered-chain samples helped preserve the initial vertical alignment (as confirmed by POM shown in Figure S8) of the liquid crystal molecules and promoted more effective reorientation under an applied electric field. As a result, these samples exhibited stronger light scattering in the on state and therefore lower transmittance. Moreover, according to the SEM images of E-8OCB and E-10OCB, the polymer fibers in the E-8OCB sample were finer and more densely distributed. This denser polymer network enhanced the anchoring effect on the liquid crystal molecules to some extent, requiring a stronger electric field to induce molecular reorientation and thereby resulting in the significantly higher driving voltage observed for the E-8OCB sample.

As shown in Figure 2d, the peel strength of the E-nOCB-based samples was further evaluated. The odd-numbered-chain samples exhibited higher peel strength than the even-numbered-chain samples. This behavior may be attributed to the looser and more disordered network structure formed by the odd-numbered chains, which increases the effective contact area with the glass substrate. In addition, when the chain parity remained unchanged, the peel strength increased with increasing chain length. Specifically, for samples with the same chain parity, increasing the chain length can enhance intermolecular interactions through stronger van der Waals forces and may also promote chain entanglement, thereby increasing the peel strength. Based on the above results, the E-10OCB sample exhibited both favorable electro-optical performance and relatively high peel strength. Therefore, it was selected for further in-depth study and application exploration.

First, the effect of the monoepoxy monomer on the performance of the PSLC films was investigated at a fixed total content of 10 wt% for the diepoxy monomer E6M and the monoepoxy monomer E-10OCB. The compositions and polymerization conditions of these samples are listed in Table 1.

**Figure 3:**
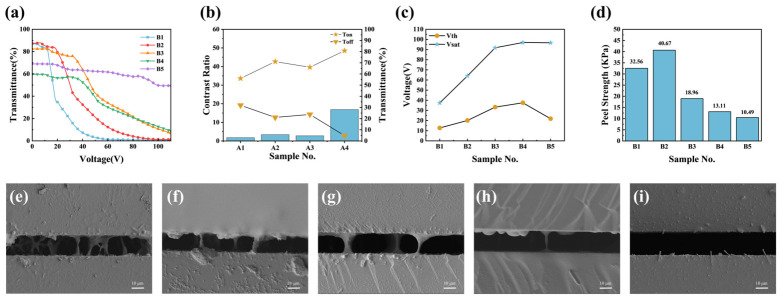
Electro-optical and mechanical performance when the total content of E6M and E-10OCB was 10 wt%: (**a**) transmittance-voltage curves, (**b**) contrast ratios and transmittance, (**c**) driving voltage, and (**d**) peel strength of Samples B1-B5; (**e**–**i**) SEM images of (**e**) B1, (**f**) B2, (**g**) B3, (**h**) B4, and (**i**) B5. All the scale bars are 10 μm.

As shown in Figure 3a,b, when the total content of E6M and E-10OCB was fixed at 10 wt%, the electro-optical performance and contrast ratio of the samples gradually decreased with increasing E-10OCB content. When the E6M:E-10OCB = 6:4, the transmittance in the on state began to increase, indicating that the ability of the liquid crystal molecules to reorient under the external electric field was markedly weakened. When the ratio was further changed to 4:6, the transmittance in the off state also decreased significantly. As a result of the combined changes in the on and off states, the contrast ratio dropped markedly.

Combined with the SEM images in Figure 3e–i, it can be seen that, with increasing E-10OCB content, the polymer morphology evolved from a network structure to columnar structures and finally to a state in which the polymer fibers gradually disappeared. This can be understood as follows: When the total polymer content remained constant and the proportion of the monofunctional monomer was relatively low, the difunctional monomer dominated the polymerization process, leading to a stronger tendency to form a continuous and dense polymer network. As the proportion of the monofunctional monomer increased, the crosslinking density of the system decreased, and the phase-separation length scale between the polymer and liquid crystal phases increased. As a result, the polymer-rich phase gradually coarsened and, under the combined effects of liquid crystal alignment and interfacial constraints, evolved into columnar structures. However, when the proportion of the monofunctional monomer became too high, the number of effective crosslinking points was insufficient to maintain a stable three-dimensional framework, causing the network or columnar structures to gradually weaken or even disappear.

As shown in Figure 3d, the mechanical properties of the samples first increased and then decreased as the E6M/E-10OCB ratio decreased. This trend can be explained by the evolution of the polymer morphology in Samples B1-B5, as shown in Figure 3e–i. At low E-10OCB content, increasing the amount of E-10OCB enhanced the lateral connectivity of the polymer network and improved its adhesion to the glass substrate, while the polymer network still maintained a relatively strong tendency to form a connected structure. As a result, the peel strength increased. However, with a further increase in E-10OCB content, although the interfacial adhesion was further improved, the polymer network became excessively sparse, resulting in a decrease in the overall peel strength.

Further, the effect of the E-10OCB on the performance of the PSLC films was investigated at a fixed total polymer content of 15 wt%. The compositions and polymerization conditions of these samples are listed in Table 1.

**Figure 4:**
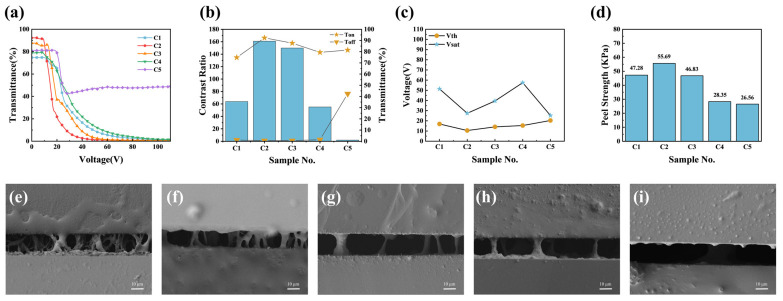
Electro-optical and mechanical performance when the total content of E6M and E-10OCB was 15 wt%: (**a**) transmittance-voltage curves, (**b**) contrast ratios and transmittance, (**c**) driving voltage, and (**d**) peel strength of Samples C1-C5; (**e**–**i**) SEM images of (**e**) C1, (**f**) C2, (**g**) C3, (**h**) C4, and (**i**) C5. All the scale bars are 10 μm.

As shown in Figure 4a,b, when the total content of E6M and E-10OCB was fixed at 15 wt%, the contrast ratio of the samples first increased and then decreased with increasing E-10OCB content. The maximum contrast ratio was obtained when the E6M:E-10OCB = 12:3. With a further increase in E-10OCB content, the contrast ratio continuously decreased. By the time the composition reached Sample C5, the electric field could not effectively drive the reorientation of the liquid crystal molecules.

Combined with the SEM images in Figure 4e–i, this behavior can be attributed to the evolution of the polymer morphology. The introduction of a small amount of E-10OCB helped make the polymer network more regular. However, as the E-10OCB content increased further, the polymer network became progressively sparser. Eventually, the system no longer formed a stable fibrous network but instead produced fragile fibers that could be readily disrupted by cyclohexane treatment.

This further explains the variation in driving voltage shown in Figure 4c. Compared with Sample C1, the polymer fibers in Sample C2 were more uniformly distributed, which to some extent facilitated the reorientation of the liquid crystal molecules under an applied electric field. However, with a further increase in E-10OCB content, the polymer fibers gradually became thicker. As a result, the anchoring force exerted by each individual fiber on the liquid crystal molecules increased, while the total number of fibers gradually decreased. By the time the composition reached Sample C5, the response of the PSLC film to the external electric field had become significantly limited.

The results shown in Figure 4d further support this interpretation. Although Samples C2 and C5 both exhibited relatively low driving voltages, the mechanical performance of C2 was clearly superior to that of C5. After C2, the mechanical properties of the PSLC films gradually deteriorated with increasing E-10OCB content.

Finally, the effect of the E-10OCB on the performance of the PSLC films was investigated at a fixed total polymer content of 20 wt%. The compositions and polymerization conditions of these samples are listed in Table 1.

**Figure 5:**
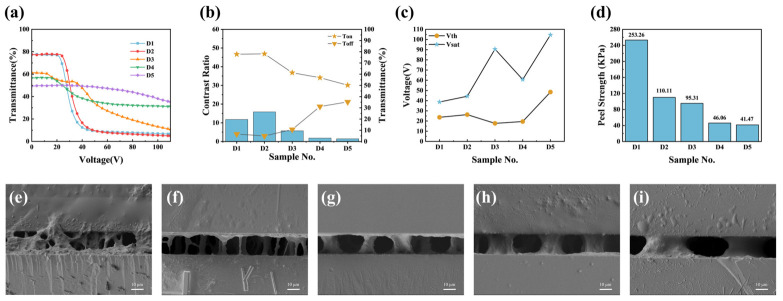
Electro-optical and mechanical performance when the total content of E6M and E-10OCB was 20 wt%: (**a**) transmittance-voltage curves, (**b**) contrast ratios and transmittance, (**c**) driving voltage, and (**d**) peel strength of Samples D1-D5; (**e**–**i**) SEM images of (**e**) D1, (**f**) D2, (**g**) D3, (**h**) D4, and (**i**) D5. All the scale bars are 10 μm.

As shown in Figure 5a,b, when the total content of E6M and E-10OCB was fixed at 20 wt%, the contrast ratio of the samples decreased with increasing E-10OCB content. Taking Sample D3 as a boundary, the contrast ratios of the samples beyond D3 were all lower than 2, indicating the limited practical applicability. Combined with the SEM images in Figure 5e–i, it can be seen that the introduction of E-10OCB still increased the size of the polymer fibers while weakening the tendency of the system to form a dense and continuous polymer network. Under these conditions, the introduction of E-10OCB could no longer further improve the electro-optical performance of the samples.

As shown in Figure 5c, the variation in driving voltage can also be understood from the evolution of the polymer morphology. With increasing E-10OCB content, although the individual polymer fibers became thicker, this change was no longer sufficient to compensate for the progressive sparsening of the overall polymer network. As a result, the overall anchoring effect on the liquid crystal molecules was weakened, leading to a decrease in the driving voltage. For the samples beyond D3, the field-induced reorientation of the liquid crystal molecules was already severely restricted, and the measured driving voltage could no longer reliably reflect the actual electro-optical performance of the films. The results in Figure 5d further support this interpretation. With further increasing E-10OCB content, the mechanical properties of the PSLC films could no longer be improved. Therefore, under the present composition conditions, further increasing the E-10OCB content not only failed to improve the electro-optical performance of the samples but also led to the deterioration of both the electro-optical and mechanical properties.

## 4. Conclusions

In this study, epoxy-based PSLC films were prepared through radical-promoted cationic photopolymerization using E6M and liquid-crystalline monoepoxy monomers (E-nOCB). The alkyl chain structure of E-nOCB strongly influenced the polymer morphology, electro-optical behavior, and peel strength. Even-numbered E-nOCB monomers favored the formation of more regular columnar polymer structures and higher optical contrast, whereas odd-numbered monomers produced more disordered networks with higher peel strength. Among them, E-10OCB showed a favorable balance between electro-optical performance and mechanical adhesion.

Further adjustment of the E6M/E-10OCB ratio showed that, at total polymer contents equal or below 15 wt%, a small amount of E-10OCB could improve both the electro-optical performance and peel strength of the PSLC films. The optimized sample achieved the highest contrast ratio of 160.91, while the peel strength increased from 47.28 to 55.69 kPa. However, further increasing the E-10OCB content weakened the peel strength of the polymer networks and deteriorated both the electro-optical and mechanical properties. These results provide an effective formulation strategy for optimizing epoxy-based PSLC films.

## Figures and Tables

**Table 1:** All compositions and UV processes used.

Sample	Composition	(wt%)	Polymerization Conditions
A1	E6M/E-7OCB/LC/1173/1176	15/5/76/2/2	240 mW/cm^2^, 300 s
A2	E6M/E-8OCB/LC/1173/1176	15/5/76/2/2	240 mW/cm^2^, 300 s
A3	E6M/E-9OCB/LC/1173/1176	15/5/76/2/2	240 mW/cm^2^, 300 s
A4	E6M/E-10OCB/LC/1173/1176	15/5/76/2/2	240 mW/cm^2^, 300 s
B1	E6M/E-10OCB/LC/1173/1176	10/0/86/2/2	240 mW/cm^2^, 300 s
B2	E6M/E-10OCB/LC/1173/1176	8/2/86/2/2	240 mW/cm^2^, 300 s
B3	E6M/E-10OCB/LC/1173/1176	6/4/86/2/2	240 mW/cm^2^, 300 s
B4	E6M/E-10OCB/LC/1173/1176	4/6/86/2/2	240 mW/cm^2^, 300 s
B5	E6M/E-10OCB/LC/1173/1176	2/8/86/2/2	240 mW/cm^2^, 300 s
C1	E6M/E-10OCB/LC/1173/1176	15/0/81/2/2	240 mW/cm^2^, 300 s
C2	E6M/E-10OCB/LC/1173/1176	12/3/81/2/2	240 mW/cm^2^, 300 s
C3	E6M/E-10OCB/LC/1173/1176	9/6/81/2/2	240 mW/cm^2^, 300 s
C4	E6M/E-10OCB/LC/1173/1176	6/9/81/2/2	240 mW/cm^2^, 300 s
C5	E6M/E-10OCB/LC/1173/1176	3/12/81/2/2	240 mW/cm^2^, 300 s
D1	E6M/E-10OCB/LC/1173/1176	20/0/76/2/2	240 mW/cm^2^, 300 s
D2	E6M/E-10OCB/LC/1173/1176	16/4/76/2/2	240 mW/cm^2^, 300 s
D3	E6M/E-10OCB/LC/1173/1176	12/8/76/2/2	240 mW/cm^2^, 300 s
D4	E6M/E-10OCB/LC/1173/1176	8/12/76/2/2	240 mW/cm^2^, 300 s
D5	E6M/E-10OCB/LC/1173/1176	4/16/76/2/2	240 mW/cm^2^, 300 s

## Data Availability

The original contributions presented in the study are included in the article/Supplementary Materials; further inquiries can be directed to the corresponding authors.

## Supplementary Materials

The following supporting information can be downloaded at https://www.mdpi.com/article/10.3390/polym18131675/s1, Figure S1. Synthetic routes of V-nOCB and E-nOCB monomers; Figure S2. ^1^H NMR spectra of (a) V-7OCB and (b) E-7OCB in CDCl_3_; Figure S3. ^1^H NMR spectra of (a) V-8OCB and (b) E-8OCB in CDCl_3_; Figure S4. ^1^H NMR spectra of (a) V-9OCB and (b) E-9OCB in CDCl_3_; Figure S5. ^1^H NMR spectra of (a) V-10OCB and (b) E-10OCB in CDCl_3_. Figure S6. ^13^C NMR spectra of (a) E-7OCB, (b) E-8OCB, (c) E-9OCB and (d) E-10OCB in CDCl_3_; Figure S7. FTIR spectra of (a) E-7OCB, (b) E-8OCB, (c) E-9OCB, and (d) E-10OCB; Figure S8. Optical microscopy images of C2 observed (a) without polarizers and (b) under crossed polarizers with a Bertrand lens.
