# Supplementary material for: Flexible Polymer-Stabilized Liquid Crystal Films Based on Radical-Promoted Cationic Co-Polymerization of Epoxy Monomers for Smart Windows
Source: Polymers (Basel). 2026 Jul 7;18(13):1675. doi: 10.3390/polym18131675 (PMC13364476; doi:10.3390/polym18131675)
Supplement: Supplementary file 1 [file polymers-18-01675-s001.zip › polymers-4399205-supplementary.pdf]

Supporting Information

Flexible Polymer-Stabilized Liquid Crystal Films Based on Radical-Promoted Cationic Co-Polymerization of Epoxy Monomers for Smart Windows

Bingxuan Wang [1], Tianfu Zhou [1], Jiayu Li [1], Yingjie Shi [1], Meiqi Yang [1], Yuxin Qian [1], Yanzi Gao [1], Meina Yu [1], Cheng Zou [1,*], Yuanwei Chen [1,*] and Huai Yang [2]

[1] Institute for Advanced Materials and Technology, University of Science and Technology Beijing, Beijing 100083, China; d202310751@xs.ustb.edu.cn (B.W.); 18579178779@163.com (T.Z.); 13131466876@163.com (J.L.); syj0625@163.com (Y.S.); yangmeiqi32@163.com (M.Y.); qianyuxin0911@163.com (Y.Q.); gaoyanzi@ustb.edu.cn (Y.G.); yumeina@ustb.edu.cn (M.Y.)

[2] School of Materials Science and Engineering, Peking University, Beijing 100083, China; yanghuai@pku.edu.cn

* Correspondence: zoucheng@ustb.edu.cn (C.Z.); chenyw@ustb.edu.cn (Y.C.)

1. Synthesis of V-nOCB and E-nOCB monomers

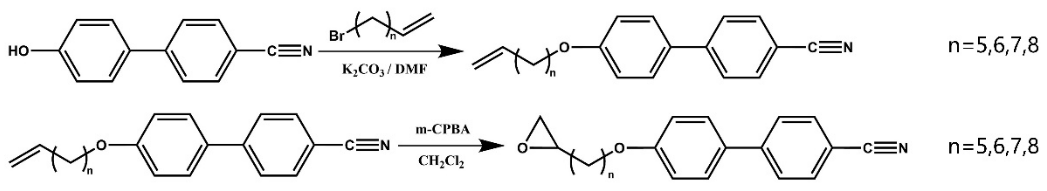

Figure S1. Synthetic routes of V-nOCB and E-nOCB monomers.

1.1 V-7OCB synthesis

A mixture of 4'-hydroxy-4-biphenylcarbonitrile (1.95 g, 0.01 mol), 7-bromo-1-heptene (2.65 g, 0.015 mol), potassium carbonate (4.14 g, 0.03 mol) and 100 mL dimethylformamide was added to a round-bottom flask equipped with a reflux condenser and magnetic stirring. The reaction mixture was refluxed at 80 °C for 24 h, then potassium carbonate was removed by filtration. 400 mL H2O and 100 mL ethyl acetate were added into the mixture, then the organic phase was collected. Most of the ethyl acetate was evaporated under reduced pressure, then the obtained crude product was purified by column chromatography with a mixture of CH2Cl2 and petroleum ether (1:3) to yield white solid V-7OCB (2.43 g, 83.3% yield).

1H NMR (400 MHz, Chloroform-d) δ 7.72–7.67 (m, 2H), 7.67–7.62 (m, 2H), 7.55–7.50 (m, 2H), 7.03–6.93 (m, 2H), 5.83 (ddt, J = 16.9, 10.2, 6.7 Hz, 1H), 5.07–4.92 (m, 2H), 4.01 (t, J = 6.5 Hz, 2H), 2.10 (dddd, J = 8.3, 6.9, 5.4, 1.8 Hz, 2H), 1.82 (dq, J = 10.7, 6.7 Hz, 2H), 1.55–1.43 (m, 4H).

1.2 V-8OCB synthesis

A mixture of 4'-hydroxy-4-biphenylcarbonitrile (1.95 g, 0.01 mol), 8-bromo-1-octene (2.86 g, 0.015 mol), potassium carbonate (4.14 g, 0.03 mol) and 100 mL dimethylformamide was added to a round-bottom flask equipped with a reflux condenser and magnetic stirring. The reaction mixture was refluxed at 80 °C for 24 h, then potassium carbonate was removed by filtration. 400 mL H2O and 100 mL ethyl acetate were added into the mixture, then the organic phase was collected. Most of the ethyl acetate was evaporated under reduced pressure, then the obtained crude product was purified by column chromatography with a mixture of CH2Cl2 and petroleum ether (1:3) to yield white solid V-8OCB (2.33 g, 76.4% yield).

1H NMR (400 MHz, Chloroform-d) δ 7.72–7.66 (m, 2H), 7.66–7.61 (m, 2H), 7.56–7.49 (m, 2H), 7.02–6.96 (m, 2H), 5.82 (ddt, J = 16.9, 10.2, 6.7 Hz, 1H), 5.01 (dq, J = 17.1, 1.7 Hz, 1H), 4.94 (ddt, J = 10.2, 2.3, 1.2 Hz, 1H), 4.00 (t, J = 6.5 Hz, 2H), 2.07 (tdd, J = 6.6, 5.3, 1.4 Hz, 2H), 1.86–1.76 (m, 2H), 1.52–1.34 (m, 6H).

1.3 V-9OCB synthesis

A mixture of 4'-hydroxy-4-biphenylcarbonitrile (1.95 g, 0.01 mol), 9-bromo-1-nonene (3.07 g, 0.015 mol), potassium carbonate (4.14 g, 0.03 mol) and 100 mL dimethylformamide was added to a round-bottom flask equipped with a reflux condenser and magnetic stirring. The reaction mixture was refluxed at 80 °C for 24 h, then potassium carbonate was removed by filtration. 400 mL

H2O and 100 mL ethyl acetate were added into the mixture, then the organic phase was collected. Most of the ethyl acetate was evaporated under reduced pressure, then the obtained crude product was purified by column chromatography with a mixture of CH2Cl2 and petroleum ether (1:2) to yield white solid V-9OCB (2.51 g, 78.7% yield).

1H NMR (400 MHz, Chloroform-d) δ 7.72–7.66 (m, 2H), 7.66–7.61 (m, 2H), 7.56–7.48 (m, 2H), 7.03–6.95 (m, 2H), 5.82 (ddt, J = 16.9, 10.2, 6.7 Hz, 1H), 5.05–4.90 (m, 2H), 4.00 (t, J = 6.5 Hz, 2H), 2.05 (tdd, J = 6.6, 5.3, 1.5 Hz, 2H), 1.81 (dq, J = 8.1, 6.6 Hz, 2H), 1.55–1.27 (m, 8H).

1.4 V-10OCB synthesis

A mixture of 4'-hydroxy-4-biphenylcarbonitrile (1.95 g, 0.01 mol), 10-bromo-1-decene (3.28 g, 0.015 mol), potassium carbonate (4.14 g, 0.03 mol) and 100 mL dimethylformamide was added to a round-bottom flask equipped with a reflux condenser and magnetic stirring. The reaction mixture was refluxed at 80 °C for 24 h, then potassium carbonate was removed by filtration. 400 mL H2O and 100 mL ethyl acetate were added into the mixture, then the organic phase was collected. Most of the ethyl acetate was evaporated under reduced pressure, then the obtained crude product was purified by column chromatography with a mixture of CH2Cl2 and petroleum ether (1:2) to yield white solid V-10OCB (2.81 g, 84.1% yield).

1H NMR (400 MHz, Chloroform-d) δ 7.71–7.66 (m, 2H), 7.66–7.61 (m, 2H), 7.57–7.48 (m, 2H), 7.03–6.95 (m, 2H), 5.82 (ddt, J = 16.9, 10.2, 6.7 Hz, 1H), 5.05–4.89 (m, 2H), 4.00 (t, J = 6.6 Hz, 2H), 2.10–2.00 (m, 2H), 1.87–1.75 (m, 2H), 1.52–1.42 (m, 2H), 1.42–1.30 (m, 8H).

1.5 E-7OCB synthesis

V-7OCB (2.91 g, 0.01 mol) was dissolved in 100 mL dichloromethane. Then, 3-chloroperoxybenzoic acid (2.58 g, 0.015 mol) dissolved in 20 mL dichloromethane was added dropwise to the stirred solution under an ice-water bath, and the reaction temperature was maintained at about 5 °C. After the addition, the reaction mixture was stirred at room temperature for 12 h. Then, 150 mL saturated sodium sulfite solution was gradually added into the reaction mixture, and the organic phase was collected after standing and phase separation. The solvent was evaporated under reduced pressure, then the obtained crude product was purified by column chromatography with a mixture of CH2Cl2 and petroleum ether (3:1) to yield white solid E-7OCB (2.31 g, 75.2% yield).

1H NMR (400 MHz, Chloroform-d) δ 7.72 (d, J = 8.5 Hz, 2H), 7.66 (d, J = 8.5 Hz, 2H), 7.59–7.51 (m, 2H), 7.05–6.97 (m, 2H), 4.04 (t, J = 6.4 Hz, 2H), 2.95 (dq, J = 6.8, 4.2, 3.1 Hz, 1H), 2.79 (dd, J = 5.0, 4.0 Hz, 1H), 2.51 (dd, J = 5.0, 2.7 Hz, 1H), 1.92–1.81 (m, 2H), 1.70–1.52 (m, 6H).

13C NMR (101 MHz, Chloroform-d) δ 132.58, 128.35, 127.10, 119.12, 115.09, 67.93, 52.25, 47.10, 32.42, 29.16, 25.93, 25.81.

1.6 E-8OCB synthesis

V-8OCB (3.05 g, 0.01 mol) was dissolved in 100 mL dichloromethane. Then, 3-chloroperoxybenzoic acid (2.58 g, 0.015 mol) dissolved in 20 mL dichloromethane was added dropwise to the stirred solution under an ice-water bath, and the reaction temperature was maintained at about 5 °C. After the addition, the reaction mixture was stirred at room temperature for 12 h. Then, 150 mL saturated sodium sulfite solution was gradually added into the reaction mixture, and the organic phase was collected after standing and phase separation. The solvent was evaporated under reduced pressure, then the obtained crude product was purified by column chromatography with a mixture of CH_2Cl_2 and petroleum ether (7:2) to yield white solid E-8OCB (2.41 g, 74.9% yield).

1H NMR (400 MHz, Chloroform-d) δ 7.69 (d, J = 8.5 Hz, 2H), 7.64 (d, J = 8.5 Hz, 2H), 7.57–7.48 (m, 2H), 7.03–6.95 (m, 2H), 4.01 (t, J = 6.5 Hz, 2H), 2.92 (dtd, J = 8.7, 4.1, 2.6 Hz, 1H), 2.75 (dd, J = 5.0, 4.0 Hz, 1H), 2.47 (dd, J = 5.0, 2.7 Hz, 1H), 1.88–1.77 (m, 2H), 1.59–1.40 (m, 8H).

13C NMR (101 MHz, Chloroform-d) δ 132.58, 128.35, 127.10, 119.13, 115.09, 110.08, 68.05, 52.33, 47.11, 32.42, 29.17, 29.12, 26.00, 25.95.

1.7 E-9OCB synthesis

V-9OCB (3.19 g, 0.01 mol) was dissolved in 100 mL dichloromethane. Then, 3-chloroperoxybenzoic acid (2.58 g, 0.015 mol) dissolved in 20 mL dichloromethane was added dropwise to the stirred solution under an ice-water bath, and the reaction temperature was maintained at about 5 °C. After the addition, the reaction mixture was stirred at room temperature for 12 h. Then, 150 mL saturated sodium sulfite solution was gradually added into the reaction mixture, and the organic phase was collected after standing and phase separation. The solvent was evaporated under reduced pressure, then the obtained crude product was purified by column chromatography with a mixture of CH2Cl2 and petroleum ether (4:1) to yield white solid E-9OCB (2.38 g, 70.9% yield).

1H NMR (400 MHz, Chloroform-d) δ 7.69 (d, J = 8.4 Hz, 2H), 7.64 (d, J = 8.4 Hz, 2H), 7.52 (d, J = 8.7 Hz, 2H), 7.03–6.95 (m, 2H), 4.01 (t, J = 6.5 Hz, 2H), 2.95–2.87 (m, 1H), 2.75 (t, J = 4.5 Hz, 1H), 2.47 (dd, J = 5.1, 2.7 Hz, 1H), 1.87–1.75 (m, 2H), 1.60–1.35 (m, 11H), 1.25 (d, J = 7.5 Hz, 1H).

13C NMR (101 MHz, Chloroform-d) δ 159.79, 132.58, 128.34, 127.09, 119.13, 115.10, 110.07, 68.11, 52.37, 47.12, 32.47, 29.36, 29.29, 29.19, 25.95.

1.8 E-10OCB synthesis

V-10OCB (3.33 g, 0.01 mol) was dissolved in 100 mL dichloromethane. Then, 3-chloroperoxybenzoic acid (2.58 g, 0.015 mol) dissolved in 20 mL

dichloromethane was added dropwise to the stirred solution under an ice-water bath, and the reaction temperature was maintained at about 5 °C. After the addition, the reaction mixture was stirred at room temperature for 12 h. Then, 150 mL saturated sodium sulfite solution was gradually added into the reaction mixture, and the organic phase was collected after standing and phase separation. The solvent was evaporated under reduced pressure, then the obtained crude product was purified by column chromatography with a mixture of CH2Cl2 and petroleum ether (5:1) to yield white solid E-10OCB (2.93 g, 83.9% yield).

1H NMR (400 MHz, Chloroform-d) δ 7.69 (d, J = 8.5 Hz, 2H), 7.64 (d, J = 8.5 Hz, 2H), 7.57–7.48 (m, 2H), 7.03–6.95 (m, 2H), 4.01 (t, J = 6.5 Hz, 2H), 2.95–2.87 (m, 1H), 2.75 (dd, J = 5.1, 4.0 Hz, 1H), 2.47 (dd, J = 5.1, 2.7 Hz, 1H), 1.87–1.75 (m, 2H), 1.60–1.44 (m, 6H), 1.44–1.35 (m, 4H).

13C NMR (101 MHz, Chloroform-d) δ 132.58, 128.34, 127.09, 119.13, 115.10, 68.15, 52.39, 47.13, 32.49, 29.47, 29.37, 29.27, 29.21, 26.01, 25.98.

2. Characterization of V-mOCB and E-mOCB monomers

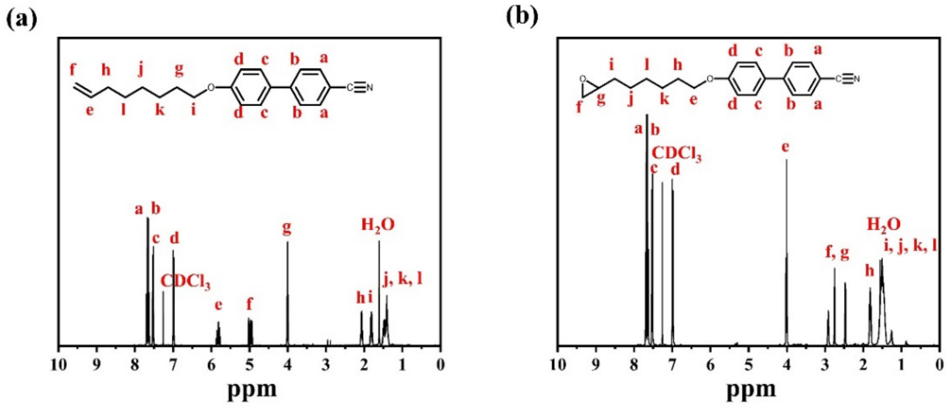

Figure S2. ^{1}H NMR spectra of (a) V-7OCB and (b) E-7OCB in $CDCl_3$.

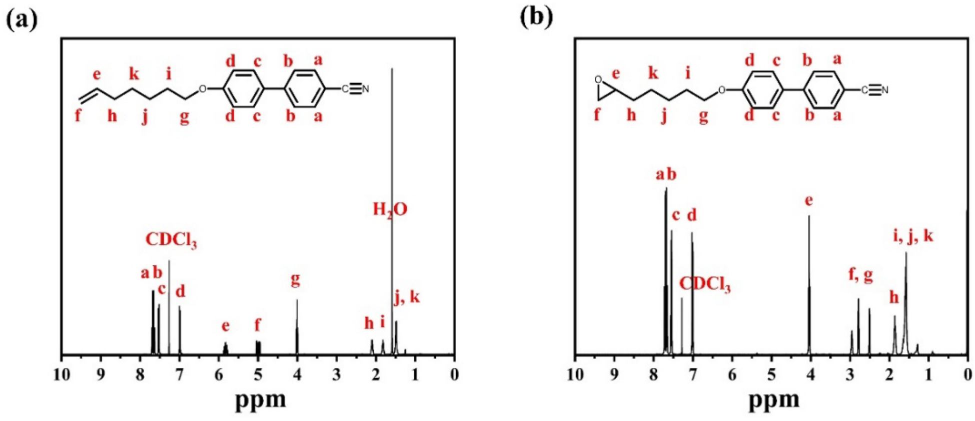

Figure S3. ^{1}H NMR spectra of (a) V-8OCB and (b) E-8OCB in $CDCl_3$.

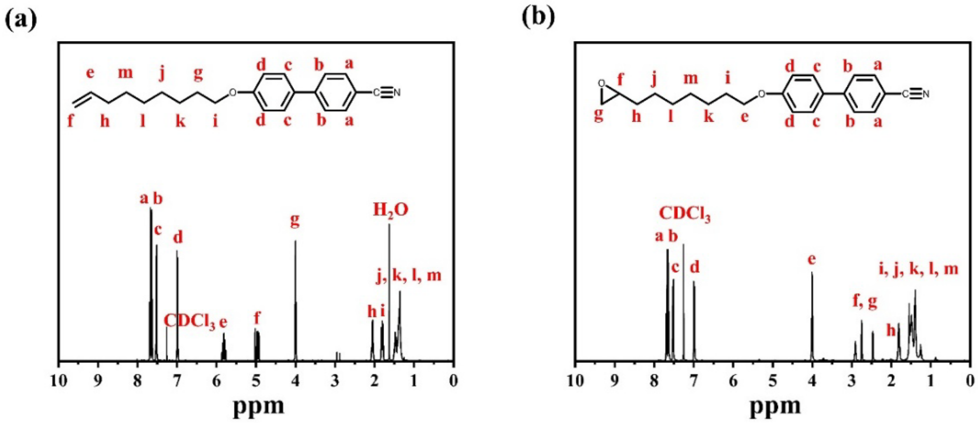

Figure S4. ^{1}H NMR spectra of (a) V-9OCB and (b) E-9OCB in $CDCl_3$.

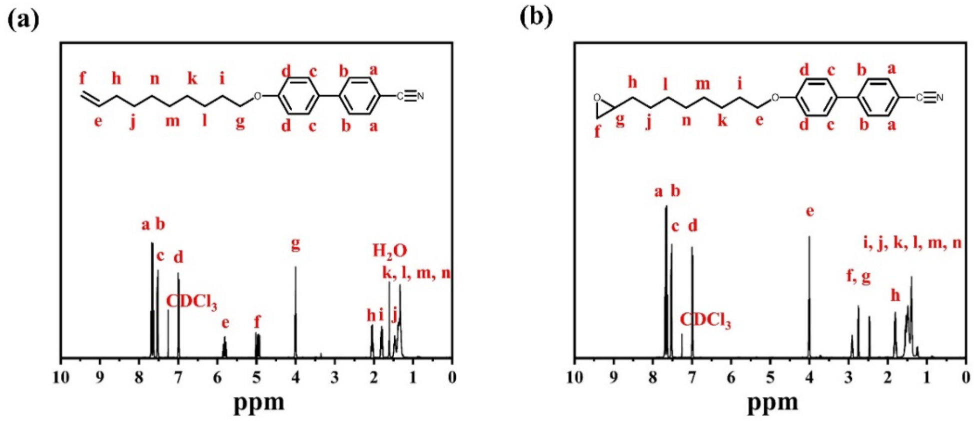

Figure S5. ^{1}H NMR spectra of (a) V-10OCB and (b) E-10OCB in $CDCl_3$.

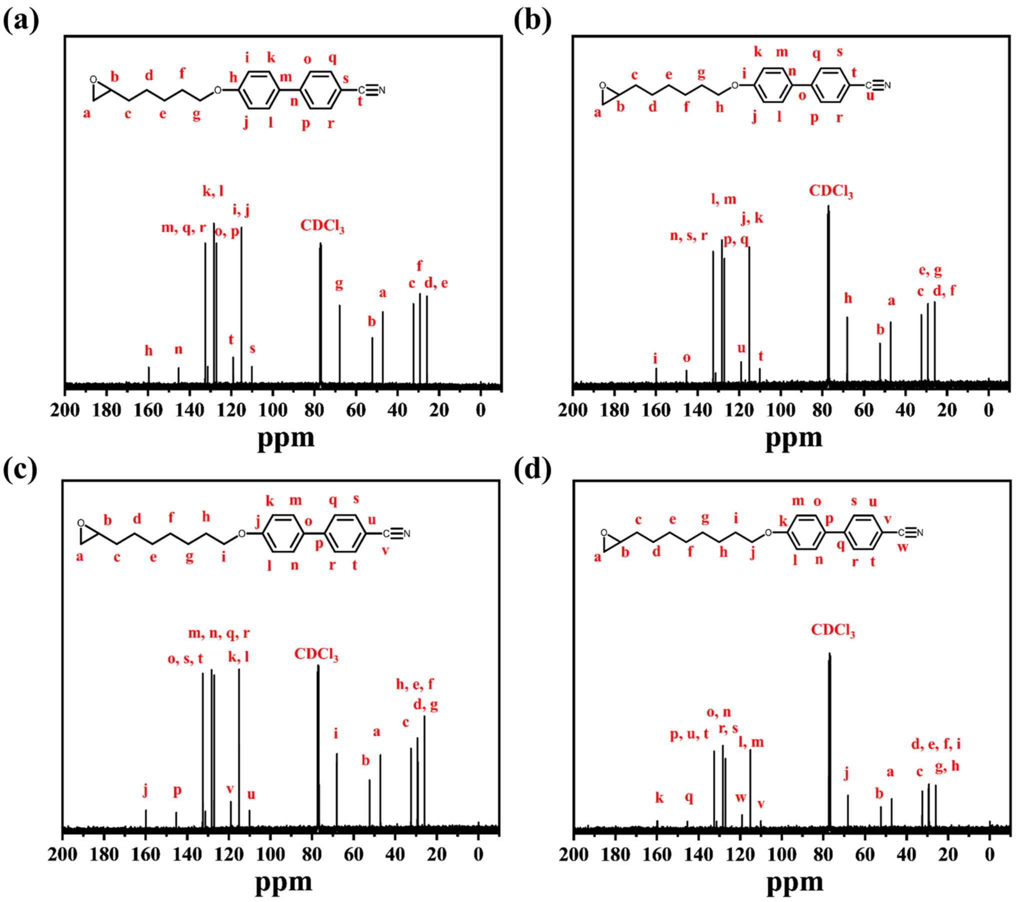

Figure S6. ^{13}C NMR spectra of (a) E-7OCB, (b) E-8OCB, (c) E-9OCB and (d) E-10OCB in $CDCl_3$.

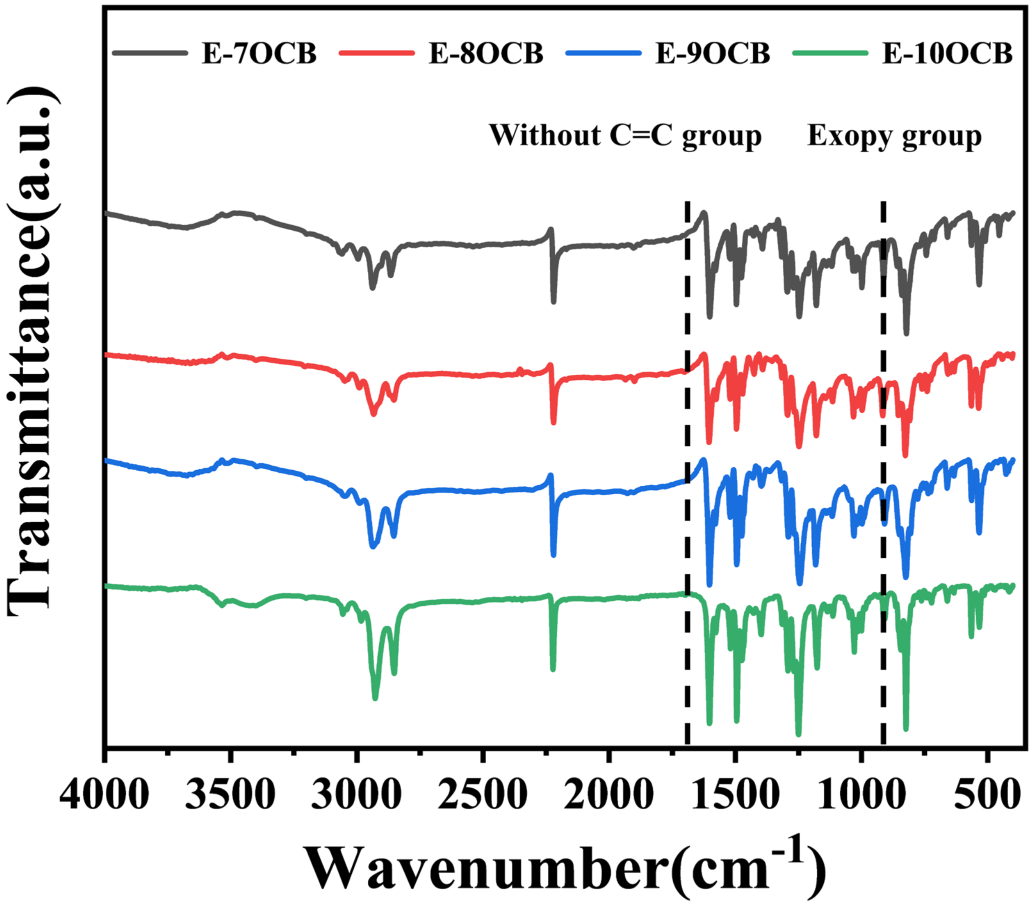

Figure S7. FTIR spectra of (a) E-7OCB, (b) E-8OCB, (c) E-9OCB, and (d) E-10OCB.

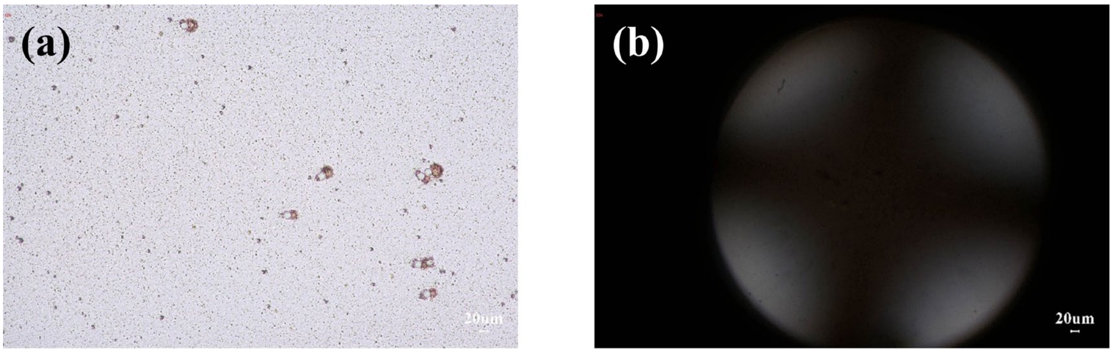

Figure S8. Optical microscopy images of C2 observed (a) without polarizers and (b) under crossed polarizers with a Bertrand lens.
